# Investigating the efficacy of novel organoclay as a rheological additive for enhancing the performance of oil-based drilling fluids

**DOI:** 10.1038/s41598-024-55246-8

**Published:** 2024-03-04

**Authors:** Ali Mahmoud, Rahul Gajbhiye, Salaheldin Elkatatny

**Affiliations:** Department of Petroleum Engineering, King Fahd University of Petroleum & Minerals, Dhahran, Saudi Arabia

**Keywords:** Energy science and technology, Materials science

## Abstract

Oil-based drilling fluids (OBDFs) are extensively used in the drilling industry due to their superior performance in challenging drilling conditions. These fluids control wellbore stability, lubricate the drill bit, and transport drill cuttings to the surface. One important component of oil-based drilling fluids is the viscosifier, which provides rheological properties to enhance drilling operations. This study evaluates the effectiveness of Claytone-IMG 400, a novel rheological agent, in enhancing the performance of OBDFs under high-pressure and high-temperature (HPHT) conditions. A comparative analysis was conducted with a pre-existing organoclay (OC) to assess the improvements achieved by Claytone-IMG 400. The OCs were analyzed using X-ray diffraction (XRD), X-ray fluorescence (XRF), scanning electron microscopy (SEM), and particle size distribution (PSD) to identify their mineral and chemical compositions, morphologies, and particle sizes. The drilling fluid density, electrical stability, sagging tendency, rheological properties, viscoelastic properties, and filtration properties were studied to formulate a stable and high-performance drilling fluid. The results confirmed that the novel OC does not affect the drilling fluid density but enhances the emulsion stability with a 9% increment compared with the drilling fluid formulated with MC-TONE. The sagging experiments showed that Claytone-IMG 400 prevented the sagging issues in both static and dynamic conditions. Also, Claytone-IMG 400 improved the plastic viscosity (PV), yield point (YP), and apparent viscosity (AV). The PV, YP, and AV were improved by 30%, 38%, and 33% increments respectively compared with the drilling fluid formulated with MC-TONE. The YP/PV ratio increased with a 6% increment from 1.12 to 1.19. Moreover, the gel strength (GS) was significantly increased, and the filtration properties were enhanced. The filtration volume was reduced by 10% from 5.0 to 4.5 cm^3^, and the filter cake thickness had a 37.5% reduction from 2.60 to 1.89 mm. The novelty of this study is highlighted by the introduction and evaluation of Claytone-IMG 400 as a new rheological additive for safe, efficient, and cost-effective drilling operations. The results indicate that Claytone-IMG 400 significantly improves the stability and performance of OBDFs, thereby reducing wellbore instability and drilling-related problems.

## Introduction

Drilling fluids are special fluids that are used in the process of drilling oil and gas wells. They serve a variety of purposes during the drilling process including lubricating and cooling the drill bit, carrying drilled cuttings to the surface, maintaining wellbore stability, and preventing formation damage or blowouts by controlling pressure imbalances between subsurface formations and the wellbore^1–5^. There are various types of drilling fluids, which can be broadly classified into two main categories namely water-based drilling fluids (WBDFs) and oil-based drilling fluids (OBDFs)^6^. Each type of drilling fluid has its unique properties and advantages, and the selection of the appropriate drilling fluid for a particular drilling operation depends on several factors, including the type of formation being drilled, the drilling depth, and the environmental regulations^7^. OBDFs are preferred for deeper and more challenging drilling operations.

OBDFs offer several advantages over WBDFs in certain drilling applications. OBDFs have better thermal stability compared to WBDFs, making them suitable for high-temperature drilling operations. This stability means that OBDFs can maintain their rheological and filtration properties under extreme temperature conditions, ensuring efficient drilling and wellbore stability^8,9^. Furthermore, OBDFs provide superior lubrication to the drill string, drill bit, and other downhole components, compared to WBDFs. This improved lubrication reduces friction, which can lead to lower rates of equipment wear, reduced torque and drag, and ultimately, extended equipment life and reduced costs^10^. Moreover, OBDFs are less reactive with shale formations than WBDFs due to their lower water content and wettability properties^11^. This reduced reactivity helps to prevent shale swelling, dispersion, or sloughing, which can lead to wellbore instability and other drilling-related issues^12^.

One of the major disadvantages of OBDFs is their potential environmental impact The use of OBDFs can lead to the release of hydrocarbons into the environment, posing a risk to aquatic life and ecosystems^13^. Also, OBDFs are generally more expensive than WBDFs due to the higher cost of the base oil and the need for additional additives to achieve the desired performance^14–16^. Moreover, if circulation losses occur during drilling, it can be challenging to stop the losses with OBDFs. Severe loss situations can be time-consuming to cure, as the drilling fluid viscosity may not increase, and the OBDF may continue opening fractures^17,18^. Furthermore, OBDFs may not be suitable for drilling certain formations, such as highly permeable formations. These formations can cause excessive fluid invasion, resulting in wellbore instability and potential formation damage^17,19^. In addition, handling and working with OBDFs may present health and safety risks to drilling personnel. The potential exposure to toxic additives and the flammability of the base oil requires strict adherence to safety protocols and the use of personal protective equipment (PPE)^20,21^.

Clay swelling in drilling operations can lead to significant challenges and potential issues. Clay swelling can lead to wellbore instability, formation damage, and potential problems such as stuck pipes, lost circulation, and well control issues^22^. When exposed to WBDFs, clays can absorb water and swell, leading to wellbore instability, decreased drilling efficiency, and potential formation damage^1^. Controlling clay swelling is crucial to ensure safe and efficient drilling operations^23^. OBDFs have proven effective in mitigating clay swelling due to their ability to prevent water penetration into the formation, thereby maintaining wellbore stability. By controlling clay swelling, OBDFs contribute to improved wellbore stability, reduced formation damage, and enhanced drilling efficiency, making them a valuable choice for challenging drilling operations^24,25^.

Drilling fluid properties play a crucial role in the efficiency, safety, and success of drilling operations. These properties can be broadly categorized into rheological properties, physical properties, and chemical properties^7,26^. Regular testing and analysis of drilling fluid properties, along with appropriate adjustments to the fluid's composition or treatment, are essential components of drilling fluid management. The selection and use of drilling fluid additives depend on several factors, including the type of formation being drilled, the drilling depth, and the environmental regulations. These additives include viscosifiers, fluid loss control agents, weighting agents, thinners, and lost circulation materials (LCMs)^27^. Proper selection and use of additives can help optimize drilling fluid performance and minimize the risk of formation damage and other problems during drilling operations.

Organoclays (OCs) are a type of clay mineral that has been modified with organic compounds to improve their performance. The modification process involves exchanging the cationic particles on the surface of the clay with an organic compound, typically quaternary ammonium compounds^28,29^. The resulting OC has unique properties that make it ideal for use in OBDFs^30^. One major advantage is its ability to increase viscosity and suspension properties while maintaining low shear rates which helps prevent cuttings from settling within the wellbore thereby improving hole stability. Other benefits include improved filtration control & rheological stability which helps enhance overall efficiency during the drilling operation^31,32^. OCs are used as additives to help control rheological properties and improve the drilling fluid performance. They are particularly useful in OBDFs and synthetic-based drilling fluids (SBDFs) due to their ability to adsorb and stabilize oil droplets and other non-polar substances within the fluid^32–37^.

Claytone-IMG 400 is a type of OC specifically designed for use as a rheological additive in OBDFs and SBDFs. It is a chemically modified smectite clay, typically derived from bentonite, treated with a quaternary ammonium compound through ion exchange. This modification enhances the clay's affinity for nonpolar organic compounds and improves its rheological properties. The specific gravity of Claytone-IMG 400 is 1.6 g/cm^3^, the bulk density is 30 lb/ft^3^, and is supplied by BYK company.

In this study, Claytone-IMG 400 is introduced as a new rheological additive to formulate drilling fluids that meet the desired performance criteria and address specific drilling challenges. It is also aimed at improving the performance of OBDFs. Important drilling fluid characteristics, represented by the drilling fluid density, emulsion stability, rheological properties, viscoelastic properties, filtration behavior, and static and dynamic sag tests were addressed and compared with an existing OC (MC-TONE) used in a drilling fluid service company. Claytone-IMG 400 exhibits enhanced affinity for nonpolar organic compounds and improved rheological properties. It offers various benefits in drilling fluid applications, including rheological control, emulsion stabilization, filtration control, and shale stabilization.

## Materials

**Figure 1:**
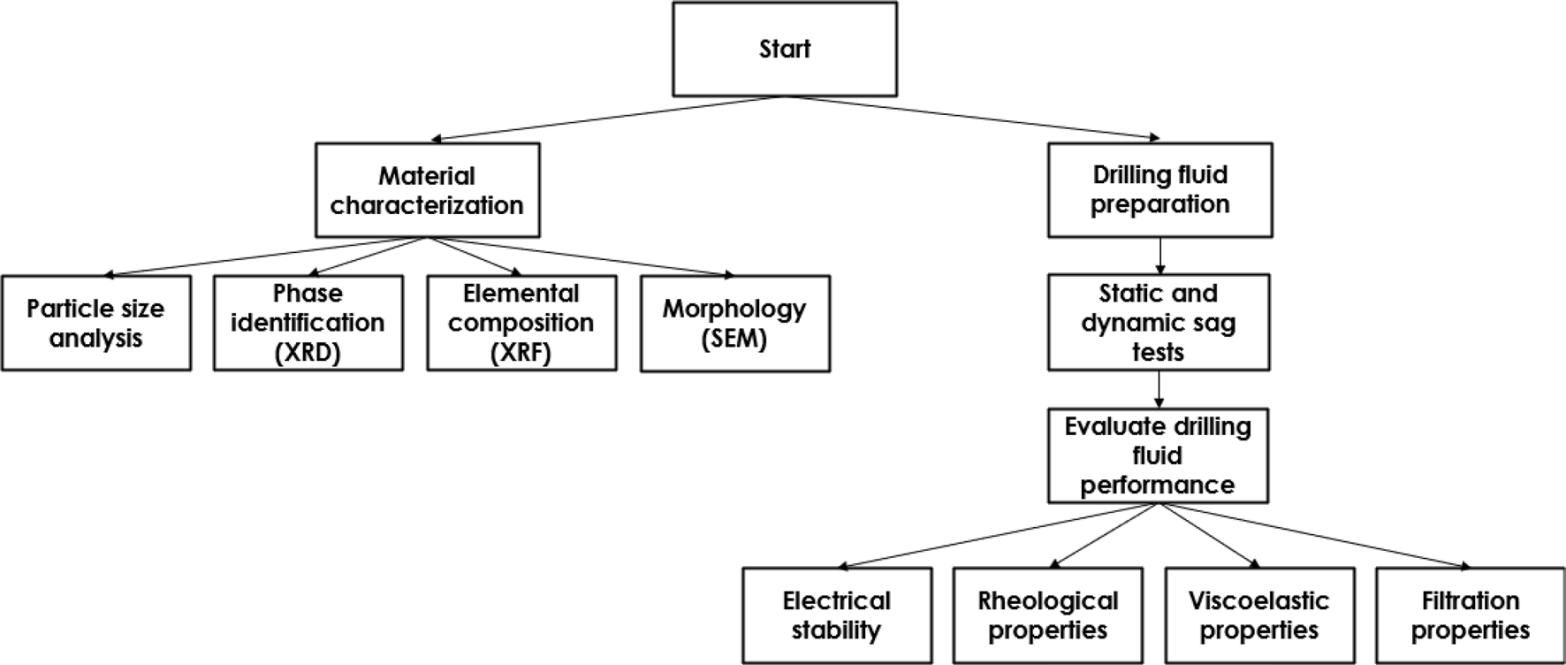
Experimental methodology.

A Hamilton Beach mixer was used to prepare two invert emulsion samples in ambient conditions. The applied field formulation is shown in Table 1 listing the practical additives with the quantities in pounds per barrel, the mixing time, and the function of each component. The mixing was done at ambient conditions and the rotational speed was 10000 RPM. The additives were mixed sequentially to control the viscosity, alkalinity fluid loss, and filter cake formation. Diesel was obtained from a local gas station while the additives were provided by a drilling fluid service provider. The experimental methodology for performing this study is summarized in Fig. 1.

**Table 1:** Drilling fluid formulation used in this study.

Additive	Quantity (ppb)	Mixing time (mins)	Function
Diesel	154.46	–	Continuous phase
Primary emulsifier	8	10	Emulsion stabilization
Secondary emulsifier	6	5	Enhance emulsion stability
Lime	6	5	Alkalinity control
CaCl_2_ brine (25% weight)	65.53	15	Dispersed phase/shale stabilization
Organoclay	7	10	Viscosifier
Organophilic lignite	8	10	Fluid loss control
Barite	377	10	Weighting material

## Experimental work

The following procedure was performed in order to evaluate the effect of using Claytone-IMG 400 on the drilling fluid properties:

1. Prepare the drilling fluid mixture at ambient temperature.
2. Measure the density and electrical stability at ambient temperature.
3. Perform the static sag test at 275 °F and 500 psi differential pressure under vertical and inclined (45°) conditions.
4. Perform the dynamic sag test using the sag shoe and the rheometer at 150 °F and 100 RPM.
5. Perform the amplitude sweep test at 275 °F and 500 psi differential pressure to determine the linear elastic region of the fluid.
6. Perform the angular frequency test at 275 °F and 500 psi differential pressure to determine the storage modulus of the invert emulsion drilling fluid.
7. Perform the time sweep test at 275 °F and 500 psi differential pressure to determine the fluid's viscosity changes over time and assesses its thixotropic behavior.
8. Measure the rheological properties at 275 °F and 500 psi differential pressure.
9. Measure the filtration properties at 275 °F and 500 psi differential pressure.

### Material characterization

Both OCs used in this study were characterized to understand the mineral compositions, elemental compositions, and morphologies by particle size distribution (PSD), X-ray diffraction (XRD), X-ray fluorescence (XRF), and scanning electron microscopy (SEM).

### Density and electrical stability

The drilling fluid weight and electrical stability under ambient conditions were measured for the two prepared drilling fluid samples using a mud balance and an electrical stability tester, respectively. The recommended electrical stability value is  > 400 Volt^38^. Monitoring and controlling the density of drilling fluids is essential for maintaining safe and efficient drilling operations. The stability of the emulsion is crucial for maintaining appropriate rheological properties, minimizing fluid loss, and ensuring overall drilling fluid performance.

### Sagging tests

Sagging tests are conducted on OBDFs to determine the stability and suspension properties of the fluid. Sagging is settling denser solids within the drilling fluid, which can lead to potential issues such as stuck pipes, lost circulation, and well control problems^39,40^. By addressing sagging issues in OBDFs, operators can minimize the associated risks and maintain efficient drilling operations.

#### Static sagging tests

This test measures the settling of solids in a fluid sample after a certain period of static conditions. The static test was conducted at vertical and vertical and inclined 45° positions since the solid sagging problems occur in both vertical and deviated wells^41^. The static sagging test was performed by subjecting the drilling fluid in an aging cell to 275 °F temperature and 500 psi pressure for a period of 24 h. The weights of 10 cm^3^ of the fluids from the upper and bottom parts of the aging cell were measured to calculate the sag factor using Eq. (1). The recommended sag factor is 0.50–0.53^42^.

1 $$SF=\frac{{\rho }_{bottom}}{{\rho }_{bottom}+{\rho }_{top}}$$

#### Dynamic sagging tests

To simulate the dynamic conditions experienced in a wellbore, the dynamic sag test was conducted at 150 °F and atmospheric pressure using a viscometer rotated at 100 rpm for 30 min. The recommended viscometer sag shoe test (VSST) value is 0–1^43^.

The following procedure was performed for the dynamic sag test^44^:

1. Insert the sag shoe into the thermocup and put it together on the viscometer plate.
2. Pour the drilling fluid inside the thermocup and raise it until the upper surface touches the lower part of the viscometer sleeve. Then lower the cup around 7 mm.
3. Heat the 140 mL drilling fluid with the sag shoe to 150 °F.
4. Set the viscometer at 100 rpm and start a 30 min timer.
5. Using the syringe with the cannula, extract a 10 mL sample and record the weight of the drilling fluid-filled syringe (W_1_).
6. Stop the viscometer after 30 min and take another sample of 10 mL.
7. Record the weight of the drilling fluid-filled syringe (W_2_).
8. Calculate the VSST using Eq. (2).
  2 $$VSST=0.833 {\text{x}} \left({W}_{2}-{W}_{1}\right)$$

### Amplitude, frequency, and time sweep tests

Amplitude, frequency, and time sweep tests are rheological tests performed on drilling fluids to understand their flow behavior and stability under different conditions. These tests are crucial for predicting the performance of the drilling fluid under the varying conditions of a drilling operation. Anton-Paar rheometer was used to conduct oscillatory amplitude, frequency, and time sweep tests. Conducting amplitude, frequency, and time sweep tests on OBDFs helps operators better understand the fluid's rheological behavior and make necessary adjustments to the fluid formulation to optimize suspension properties, hole cleaning, and wellbore stability.

#### Amplitude sweep tests

This test evaluates the linear viscoelastic region (LVE) of the drilling fluid by varying the applied shear strain while maintaining a constant frequency. The LVE is the range where the fluid exhibits a linear relationship between the applied stress and the resulting strain. The point at which the fluid's response deviates from linearity is considered its yield point, which is a measure of the fluid's ability to carry cuttings. The test helps to identify the optimal range of shear stress or strain for subsequent frequency and time sweep tests. Oscillatory amplitude tests were conducted to study the effect of using the novel OC on the storage modulus G' and loss modulus G''. The amplitude test was conducted at a fixed frequency of 10 rad/s and the range of shear strains from 0.01 to 100% to determine the region of linear viscoelastic and stability.

#### Frequency sweep tests

In a frequency sweep test, the frequency of the applied oscillatory shear stress is varied while keeping the shear strain constant, usually within the LVE. The test provides information about the fluid's viscoelastic behavior over a range of frequencies, which represent different shear rates that the fluid may experience during drilling operations. The frequency test was carried out at different frequencies (0.1–100 rad/s) and constant shear strain was selected from the linear range to study the elasticity behavior of the inner structure.

#### Time sweep tests

The time sweep test monitors the changes in the fluid's rheological properties over time under constant amplitude and frequency conditions. This test is particularly useful for evaluating the stability of the fluid's viscoelastic properties during aging or as a function of time under specific shear conditions. This test can provide information about the fluid's thixotropic behavior (time-dependent shear thinning property) and its ability to maintain its properties over time. The time sweep test applies an oscillatory shear stress or strain to the fluid sample at a constant amplitude and frequency. The rheometer will record the fluid's viscoelastic properties, such as storage modulus G' and G", at regular time intervals during the test for 60 min.

### Rheology tests

Rheology tests are used to evaluate the flow properties of drilling fluids. These tests are important for assessing the ability of the fluid to carry cuttings to the surface, prevent formation damage, and maintain wellbore stability. The rheological properties of OBDFs were measured at 275 °F and 500 psi differential pressure using a rheometer. The plastic viscosity (PV), yield point (YP), and apparent viscosity (AV) using a Grace viscometer (model M3600) to investigate the effect of novel OC on the drilling fluid rheology compared with the drilling fluid formulated using the pre-exisiting OC. The Grace viscometer is designed to measure the rheological properties of drilling fluids, specifically their viscosity and gel strength (GS). It is commonly used in the oil and gas industry for quality control and performance evaluation of drilling fluid formulations. The Model M3600 Grace viscometer operates based on the principle of rotational viscometry. The instrument consists of a cylindrical cup containing the drilling fluid sample and a spindle that rotates at a constant speed within the fluid. As the spindle rotates, it experiences resistance from the fluid, which is measured as torque by the viscometer. The Grace M3600 viscometer offers several features and capabilities including multiple speed settings, temperature control, data display and storage, and a user-friendly interface. The GS was estimated directly from dial reading at 3 RPM after different periods (i.e., 10 s, 10 min, and 30 min). The following formulas were implemented to estimate the PV, YP, and AV:

3 $$PV={\varnothing }_{600}-{\varnothing }_{300}$$

4 $$YP={\varnothing }_{300}-PV$$

5 $$AV=\frac{{\varnothing }_{600}}{2}$$

### Filtration tests

The HPHT filtration loss test is a crucial laboratory test performed on OBDFs to evaluate their fluid loss performance under simulated downhole conditions. Fluid loss control is important to maintain wellbore stability, minimize formation damage, and ensure efficient drilling operations. The HPHT filtration loss test is conducted using a specialized HPHT filter press, which subjects the drilling fluid sample to elevated pressure and temperature conditions. A specialized HPHT filter press is a type of filtration equipment specifically designed to evaluate the filtration properties of drilling fluids under HPHT conditions. It is commonly used in the oil and gas industry to simulate downhole conditions and assess the performance of drilling fluid formulations in preventing fluid loss and maintaining wellbore stability. The HPHT filter press consists of a cylindrical cell or chamber with a filter medium that allows fluid to pass through while retaining solid particles. The drilling fluid is placed in the cell, and pressure is applied to simulate the downhole pressure experienced during drilling operations. Additionally, the system is heated to replicate the high temperatures encountered in deep wells. The test measures the volume of filtrate that passes through a filter medium under specified temperature and pressure conditions. The filtration test was performed at 275 °F and 500 psi differential pressure using filter paper as the filtration medium. The filtration volume was monitored with respect to time for 30 min, and the filter cake thickness was measured.

## Results and discussions

### Material characterization

**Figure 2:**
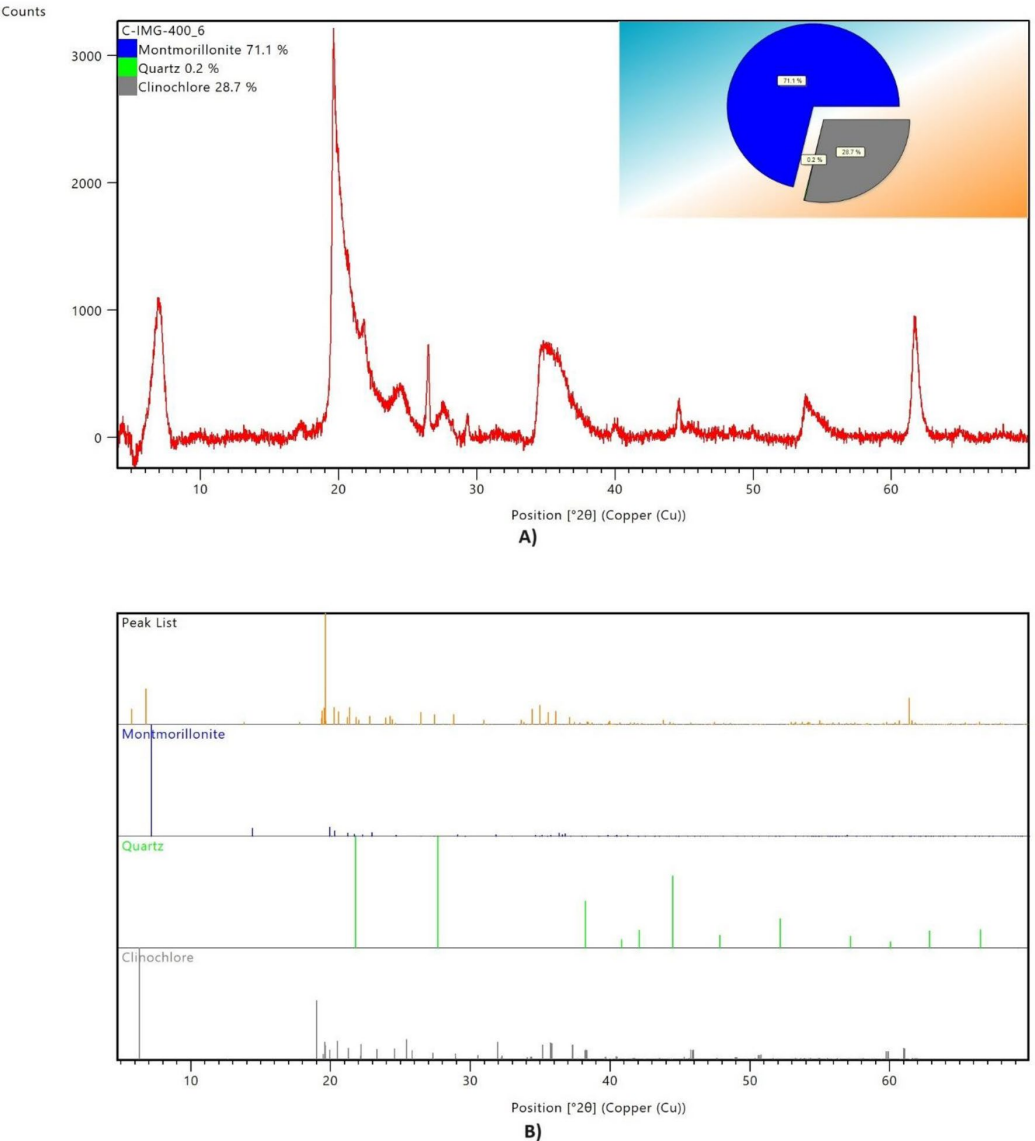
The (**A**) XRD pattern and (**B**) plot of identified phases of Claytone-IMG 400.

**Figure 3:**
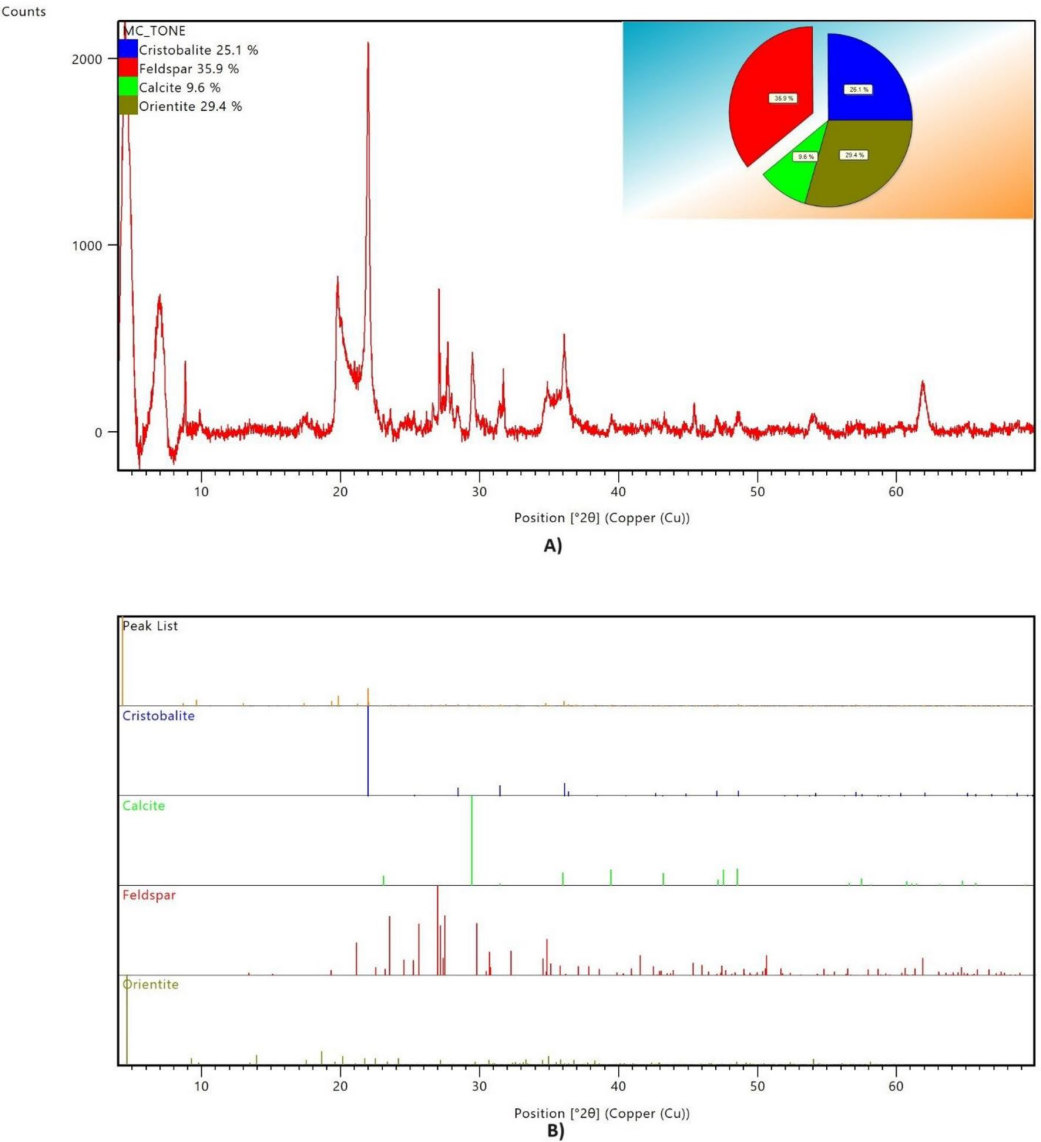
The (**A**) XRD pattern and (**B**) plot of identified phases of MC-TONE.

Figure 2 shows the XRD pattern and the plot of identified phases of Claytone-IMG 400. The main components of Claytone-IMG 400 are montmorillonite (71.1%) and clinochlore (28.7%) with a small percentage of quartz (0.2%). Figure 3 shows the XRD pattern and the plot of identified phases of MC-TONE. The main components of MC-TONE consist of feldspar (35.9%), orientite (29.4%), cristobalite (25.1%), and calcite (9.6%). Table 2 shows the chemical compositions of the two OCs used in this study. Claytone-IMG 400 is composed of silicon (47.41%), aluminum (34.86%), magnesium (10.41%), sodium (6.15%), and iron (1.11%), while MC-TONE is composed of silicon (53.67%), aluminum (14.67%), chlorine (11.84%), iron (11.09%), and calcium (7.08%).

**Table 2:** XRF analysis for the OCs used in this study.

Sample ID/Chemical composition (%)	Na	Mg	Al	Si	Fe	Cl	K	Ca
Claytone-IMG 400	6.15	10.47	34.86	47.41	1.11	–	–	–
MC-TONE		–	14.67	53.67	11.09	11.84	1.65	7.08

**Figure 4:**
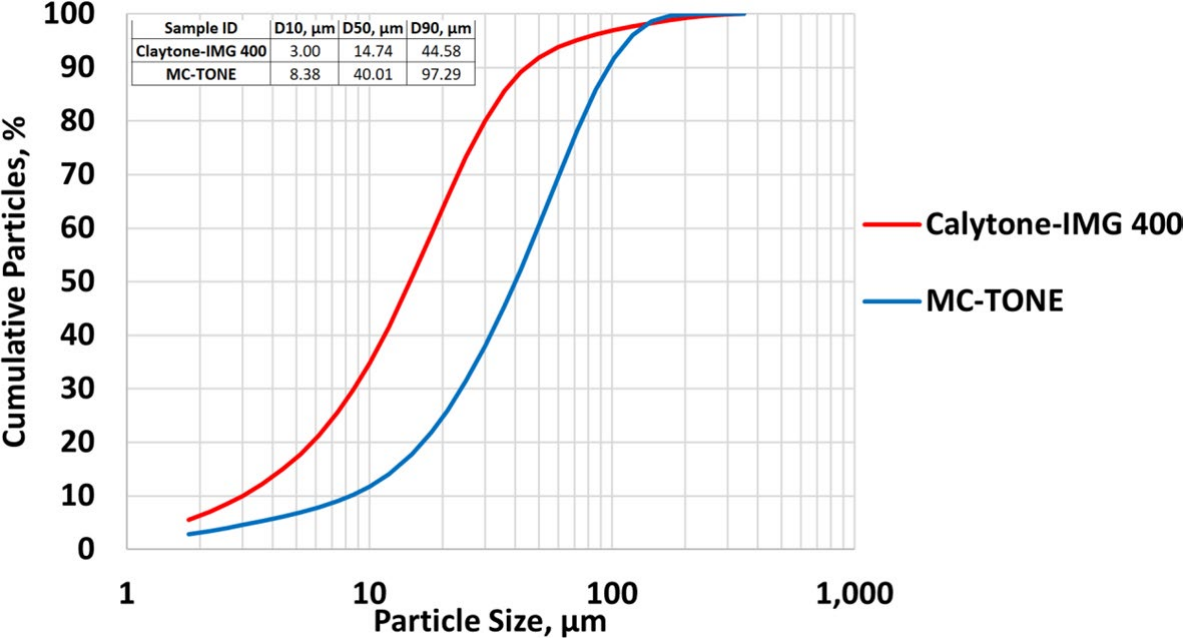
PSD for Claytone-IMG 400 and MC-TONE.

The results of the PSD shown in Fig. 4 show that the average particle size (D_50_) of Claytone-IMG 400 is 14.74 *µ*m and that of MC-TONE is 40.01 *µ*m. The fine particle size of Claytone-IMG 400 is desirable in many applications because it can lead to improved dispersion and stability within non-polar liquids like OBDFs. Claytone-IMG 400 has a small average particle size distribution, which contributes to its desirable properties such as high surface area, good dispersion, and high brightness. It is also characterized by its low abrasiveness, low moisture content, and high purity.

**Figure 5:**
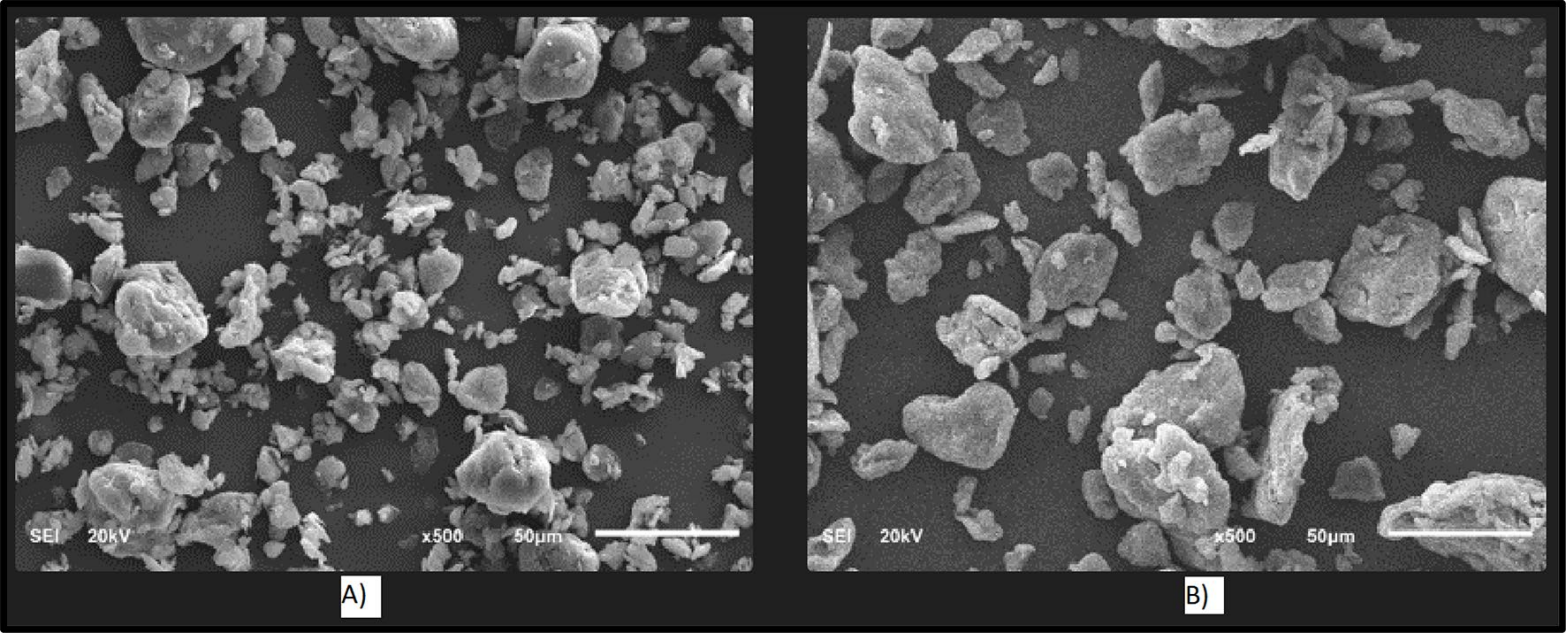
SEM images of (**A**) Claytone-IMG 400 and (**B**) MC-TONE.

The SEM images for both OCs used in this study are shown in Fig. 5. In SEM images of Claytone-IMG 400, the quaternary ammonium compounds used in surface modification treatment create distinct morphological features on the surfaces of these particles which helps them disperse better within non-polar fluids. Furthermore, it is common to observe a collection of small, irregularly shaped particles with a relatively smooth surface texture. The particles appear to be loosely packed together, forming a porous structure with many small voids and channels. This porous structure is due to the irregular shape of the particles and the way they pack together. Moreover, the particles appear as individual platelets or stacks that are held together by interactional mechanisms such as ion–dipole interaction, hydrogen bonds, acid–base reactions, charge transfer, electrostatic interaction, and van der Waals forces^28,45,46^. Also, the uniformity and small size distribution seen in SEM images suggest that Claytone-IMG 400 is well dispersed within non-polar fluids like OBDFs. Compared to Claytone-IMG 400, MC-TONE has higher irregularity and sharper particle edges which increases the sagging tendency.

### Density and electrical stability

**Figure 6:**
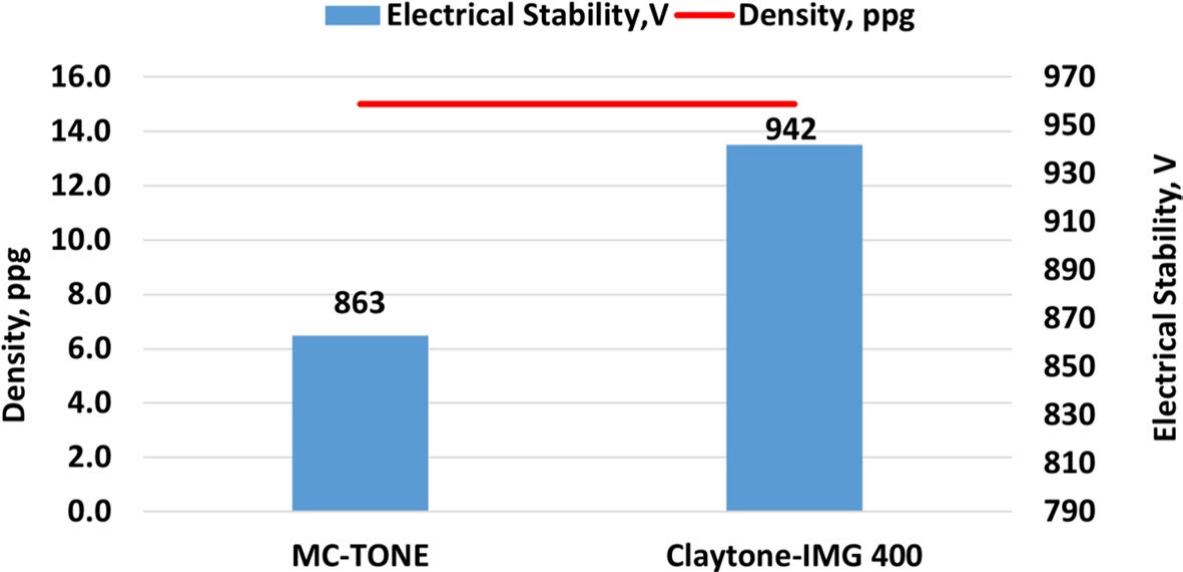
Effect of Claytone-IMG 400 on the drilling fluid density and electrical stability.

No change in the density of the drilling fluid was observed when adding Claytone-IMG 400 OC instead of MC-TONE OC as it remains unchanged at 15 ppg. Figure 6 shows that Claytone-IMG 400 enhanced the electrical stability of the invert emulsion with a 9% increment to 942 Volt compared to 863 Volt for the drilling fluid prepared with MC-TONE. This is due to its relatively small particle size and high surface area^47^. The quaternary ammonium compounds used in surface modification treatments make the clay particles more hydrophobic, which can reduce their tendency to flocculate under certain conditions^48–50^. Additionally, the small average particle size distribution of Claytone-IMG 400 contributes towards improved electrical stability by reducing sedimentation rates in suspensions when subjected to an electric field. Smaller particles tend to have less settling velocity than larger ones due to their smaller mass thus allowing them to remain suspended for longer periods^10,39^. Furthermore, Claytone-IMG 400 can reduce the affinity of water droplets for the solid surfaces present in the fluid system. This reduction in water wetting can help to minimize the contact between water and conductive solids, thereby reducing the potential for electrical conductivity and stability issues^51–53^.

### Sagging tests

#### Static sagging tests

**Figure 7:**
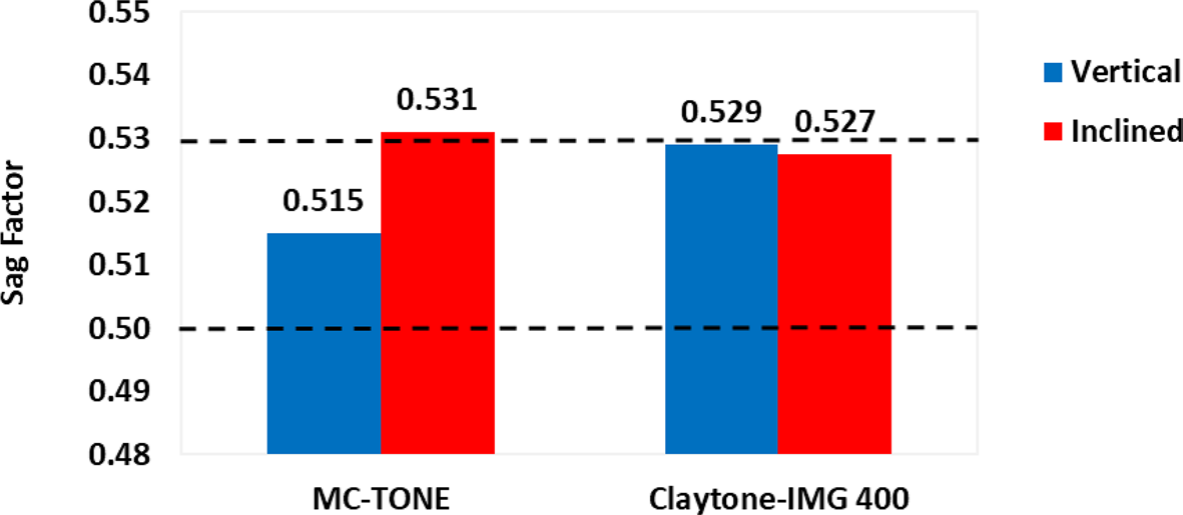
Effect of Claytone-IMG 400 on the static sag factors.

The vertical and inclined conditions sag factors for the MC-TONE/oil and Calytone-IMG 400/oil fluids are shown in Fig. 7. It is clear that Claytone-IMG 400 increased the vertical sag to 0.529 compared to the drilling fluid formulated using MC-TONE 0.515 but this increase is within the acceptable range. Furthermore, Claytone-IMG 400 decreased the sag factor from 0.531 which is higher than the recommended safe range of 0.50–0.53^40^ to 0.527. The mechanism by which Claytone-IMG 400 helps prevent static sag involves its ability to function as a rheological modifier and its impact on the suspension stability of the fluid system. Claytone-IMG 400 can impart thixotropic behavior to a fluid system, which means that the system's viscosity decreases when subjected to shear and quickly recovers when the shear is removed. Thixotropic behavior can help prevent static sag by allowing the fluid to flow more easily under shear and then rapidly increase in viscosity when at rest, preventing the settling of particles^42^. Furthermore, Claytone-IMG 400 also contributes to the yield stress of a fluid system, which is the minimum stress required to initiate flow. A higher yield stress means that the fluid system can resist the gravitational force acting on the suspended particles more effectively, helping to prevent static sag and maintain a stable suspension^40,42^. Moreover, the interaction between Claytone-IMG 400 particles and the other components in the fluid system can influence the overall suspension stability. OCs can form a network structure within the fluid, which helps to support and stabilize the suspended particles, preventing them from settling or separating^54^. Also, Claytone-IMG 400 can adsorb onto the surfaces of suspended particles, altering their surface properties and improving the overall stability of the suspension. The adsorption of OCs can help to prevent aggregation and settling of particles, which contributes to the prevention of static sag^55^.

#### Dynamic sagging tests

**Figure 8:**
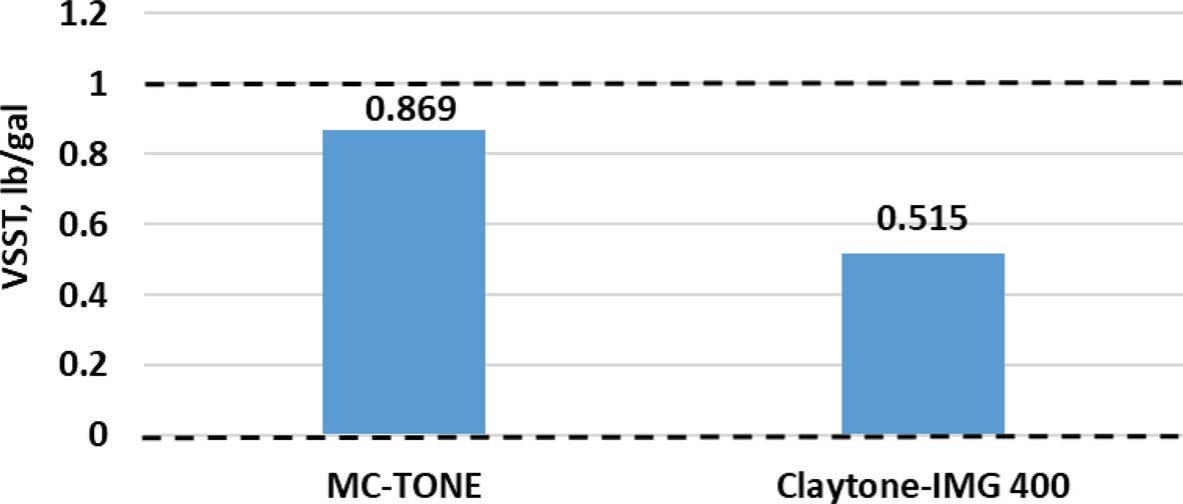
Effect of Claytone-IMG 400 on the dynamic sag factor.

The VSST sag factors for both OCs are shown in Fig. 8. The results show a reduction in the VSST value from 0.869 to 0.515 ppg. Claytone-IMG 400 can contribute to the yield stress of the fluid system, which is the minimum stress required to initiate flow. A higher yield stress helps the fluid system resist the component of gravitational force acting during dynamic motion more effectively, preventing dynamic sag and maintaining a stable suspension^40,42^. In dynamic applications, Claytone-IMG 400 can form a network structure within the fluid that supports and stabilizes the suspended particles, preventing them from settling downward under gravity during motion which influences the overall suspension stability. Moreover, adsorption can help prevent aggregation and settling of particles in dynamic applications, contributing to the prevention of dynamic sag^55^. Furthermore, it allows the fluid to flow and level out easily during application and then rapidly increase in viscosity when subjected to dynamic forces, preventing the sagging, or settling of particles due to its positive effect on the thixotropic behavior of the fluid.

### Amplitude, frequency, and time sweep tests

#### Amplitude sweep tests

**Figure 9:**
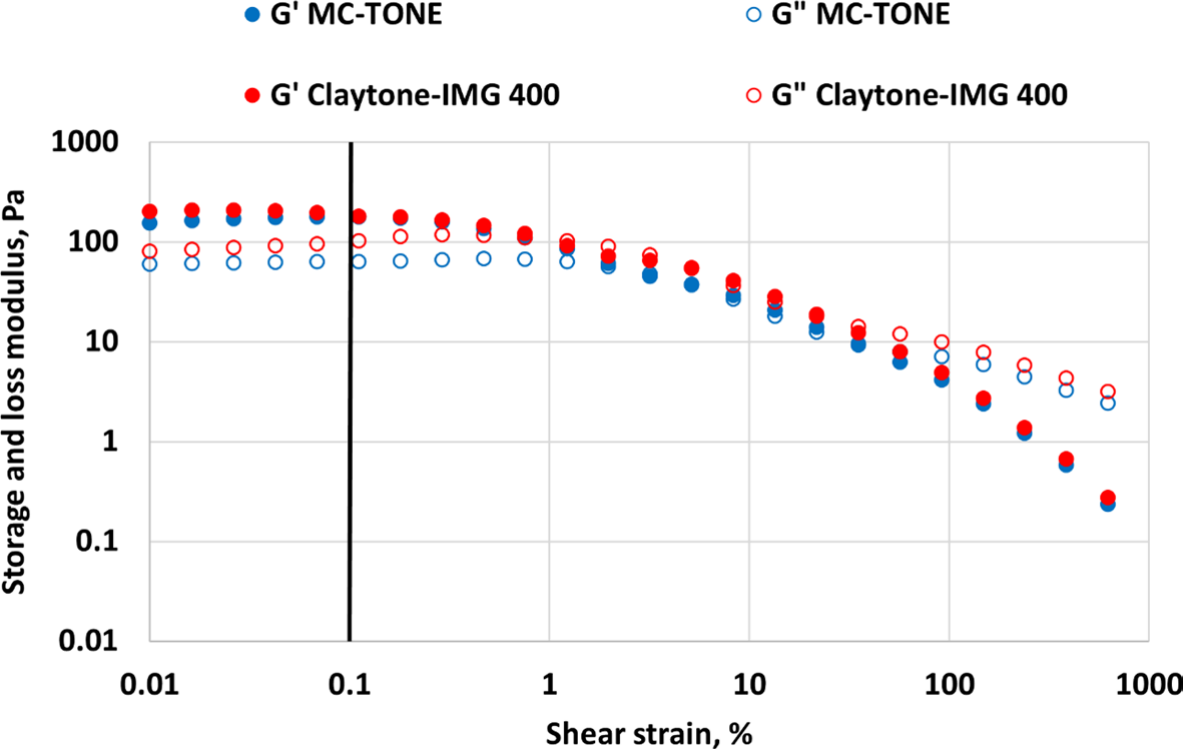
Effect of Claytone-IMG 400 on oscillatory amplitude sweep test.

The oscillatory amplitude test showed that Claytone-IMG 400 results in a small increase for both G' and G" in the LVE region that is limited to 0.1% shear strain compared to the fluid formulated using MC-TONE as shown in Fig. 9. This results in better elasticity, sag resistance, and a solid-like behavior. Claytone-IMG 400 can increase the yield stress, indicating better resistance to deformation and improved stability under applied stress. Claytone-IMG 400 can increase the elastic or solid-like behavior of a formulation, which is reflected in a higher G' value. This can be beneficial in applications where good sag resistance, thixotropic behavior, or suspension properties are desired^56,57^. Furthermore, The presence of Claytone-IMG 400 can also affect the viscous or liquid-like behavior of a formulation, as shown by changes in the G" value. An increased G" value can lead to better leveling or flow control in specific applications. Moreover, the addition of Claytone-IMG 400 can enhance the overall viscoelastic properties of a formulation. This can lead to improved performance in terms of flow control, stability, and resistance to deformation. Also, incorporating Claytone-IMG 400 into a formulation can result in increased complex viscosity, indicating a higher resistance to flow under oscillatory shear. This can be beneficial for applications that require shear-thinning behavior or improved sag resistance^56,57^.

#### Frequency sweep tests

**Figure 10:**
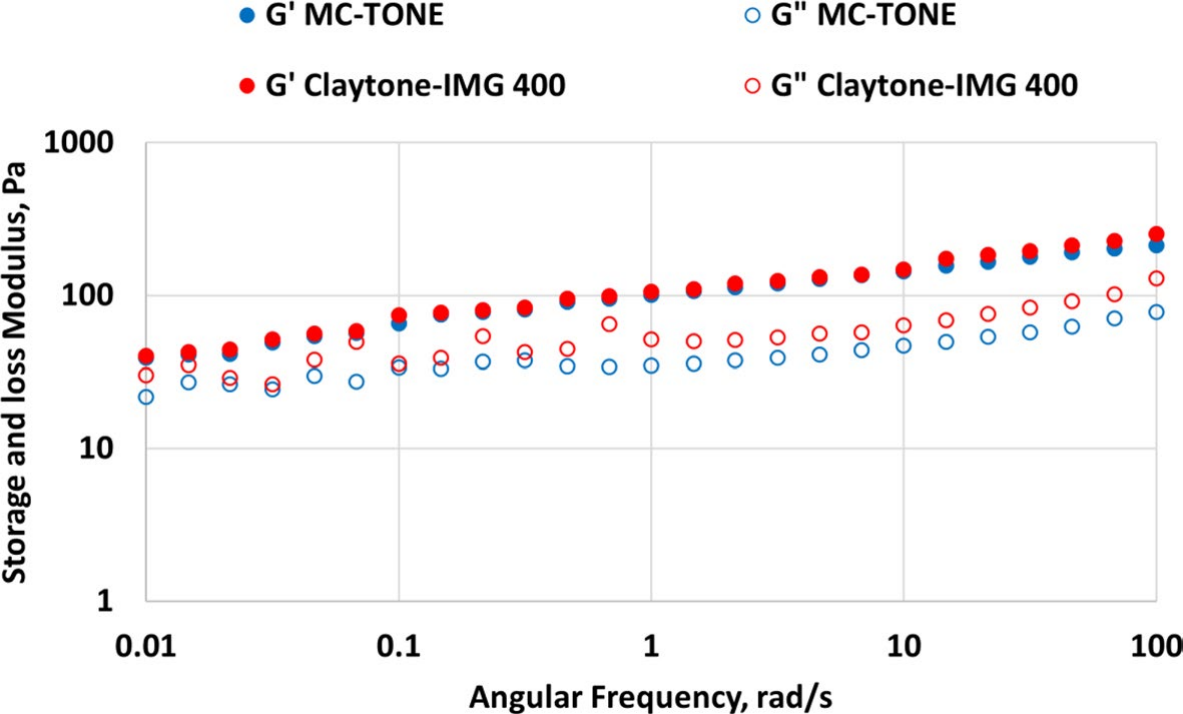
Effect of Claytone-IMG 400 on oscillatory frequency sweep test.

Figure 10 shows the results of the oscillatory frequency sweep tests for the MC-TONE/oil fluid and Claytone-IMG 400/oil fluid. When Claytone-IMG 400 is added to a drilling fluid, it has been shown to enhance both G' and G", indicating improved elasticity and viscous behavior. This means that at varying frequencies, the fluid will maintain its desirable flow properties without experiencing sagging or settling. Furthermore, Claytone-IMG 400 improves suspension properties within drilling fluids by preventing solids from settling out and causing issues such as blockages or pump wear. Also, Claytone-IMG 400 increases the material's storage modulus and improves its mechanical strength, which could be beneficial for applications that require high stiffness or resistance to deformation^58^. Moreover, Claytone-IMG 400 also increases the material's viscosity and reduces its flowability, which could be beneficial for applications that require high thixotropy or sag resistance^56^. The addition of Claytone-IMG 400 can improve the stability of a formulation by increasing its yield stress and resistance to deformation. In a frequency sweep test, this can be observed as an increase in G' at low frequencies, indicating enhanced elastic or solid-like behavior^57^.

#### Time sweep tests

**Figure 11:**
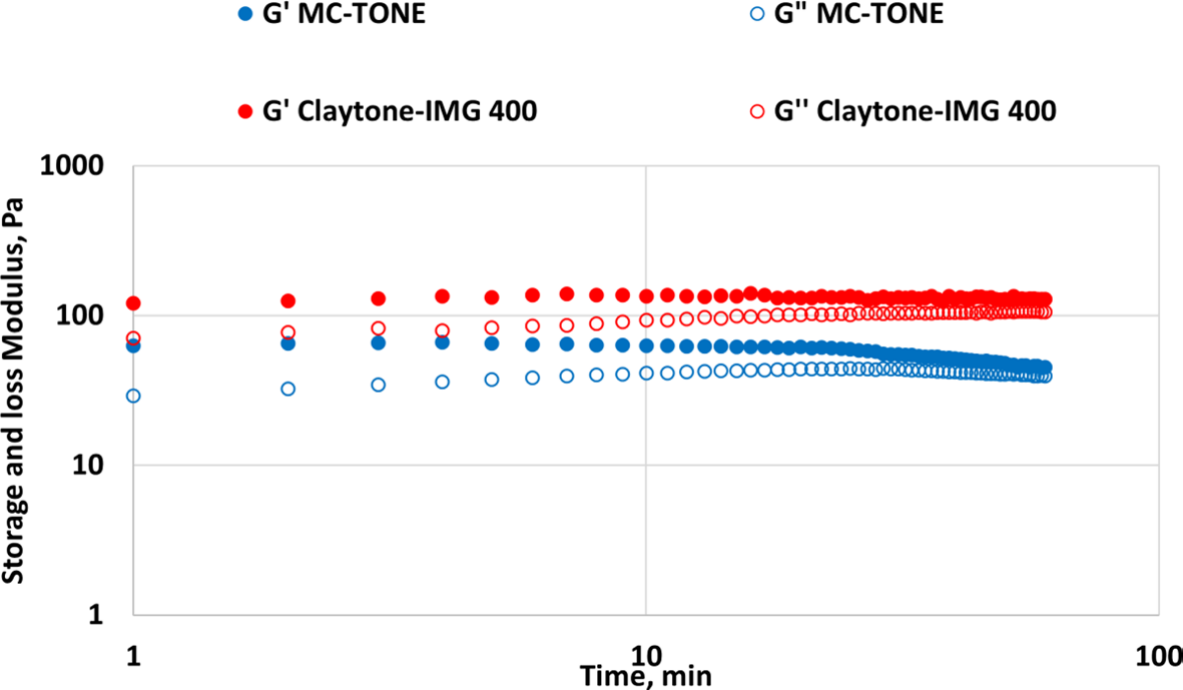
Effect of Claytone-IMG 400 on time sweep test.

The results of the time sweep tests are shown in Fig. 11. Claytone-IMG 400 increases the G' of the material, indicating improved solid-like behavior and resistance to deformation. The change in G" over time may provide insights into the material's viscosity and liquid-like behavior. Furthermore, the addition of Claytone-IMG 400 also affects the G" of the material. Moreover, Claytone-IMG 400 enhances the thixotropic behavior of a formulation, allowing it to thin under shear stress and recover its initial viscosity once the stress is removed^57^. This property can be observed through the recovery of G' and G" after a change in applied stress, demonstrating the material's ability to regain its structure over time^59^.

### Rheology tests

**Figure 12:**
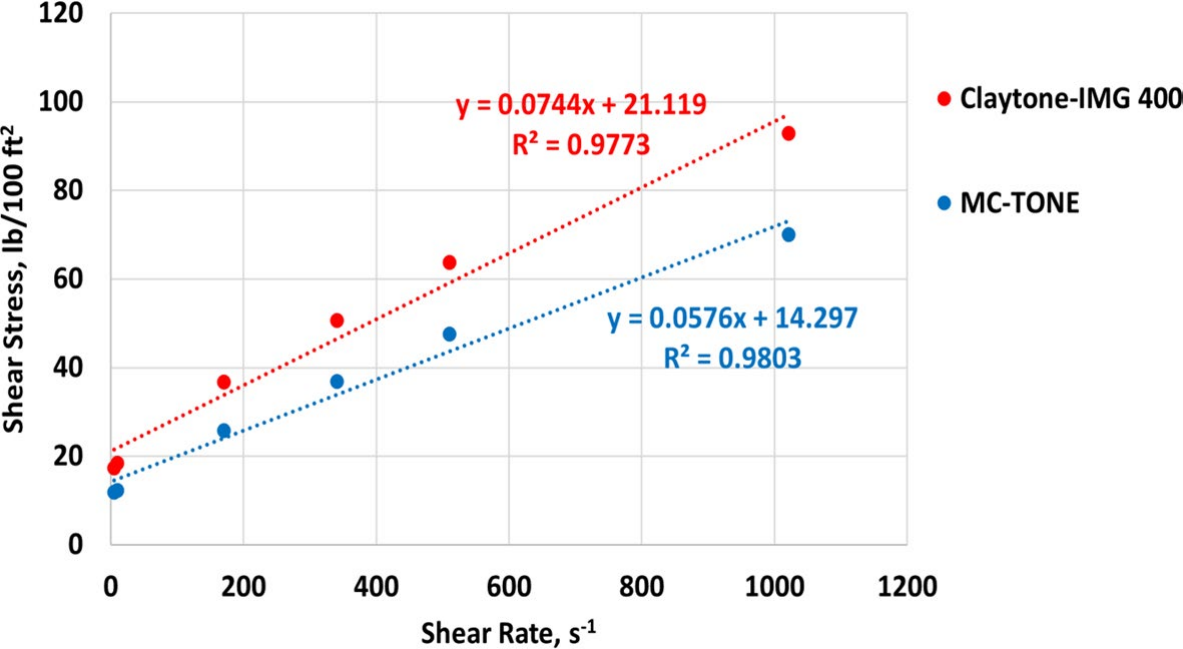
Effect of Claytone-IMG 400 on the stress–strain relationship at 275°F.

**Figure 13:**
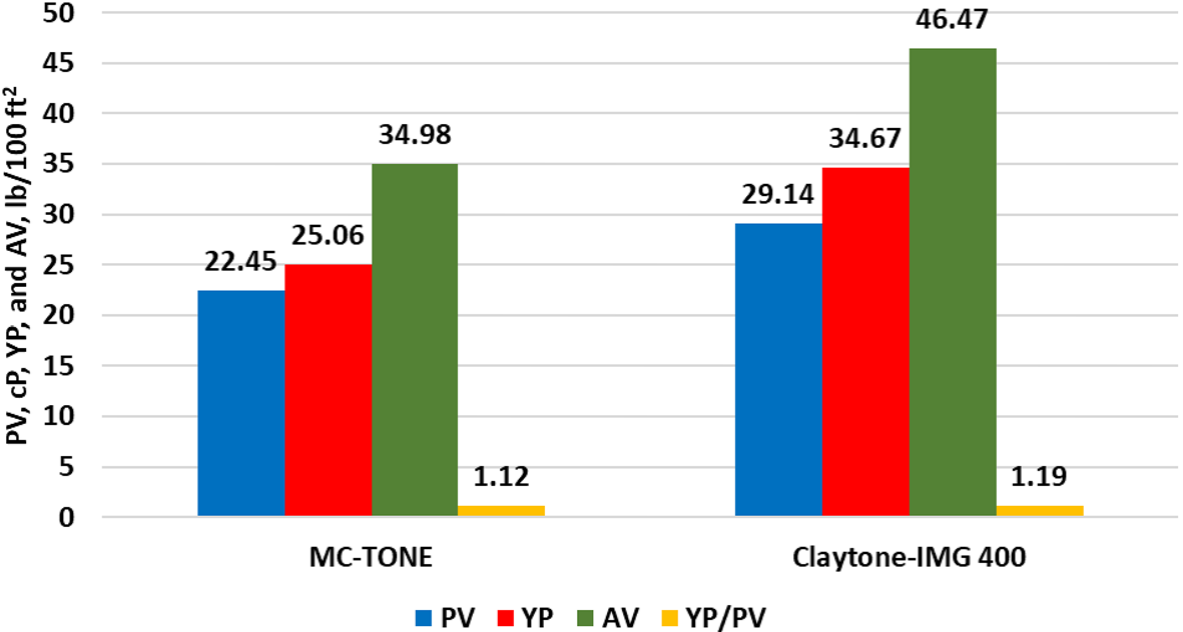
Effect of Claytone-IMG 400 on drilling fluid rheology at 275 °F.

**Figure 14:**
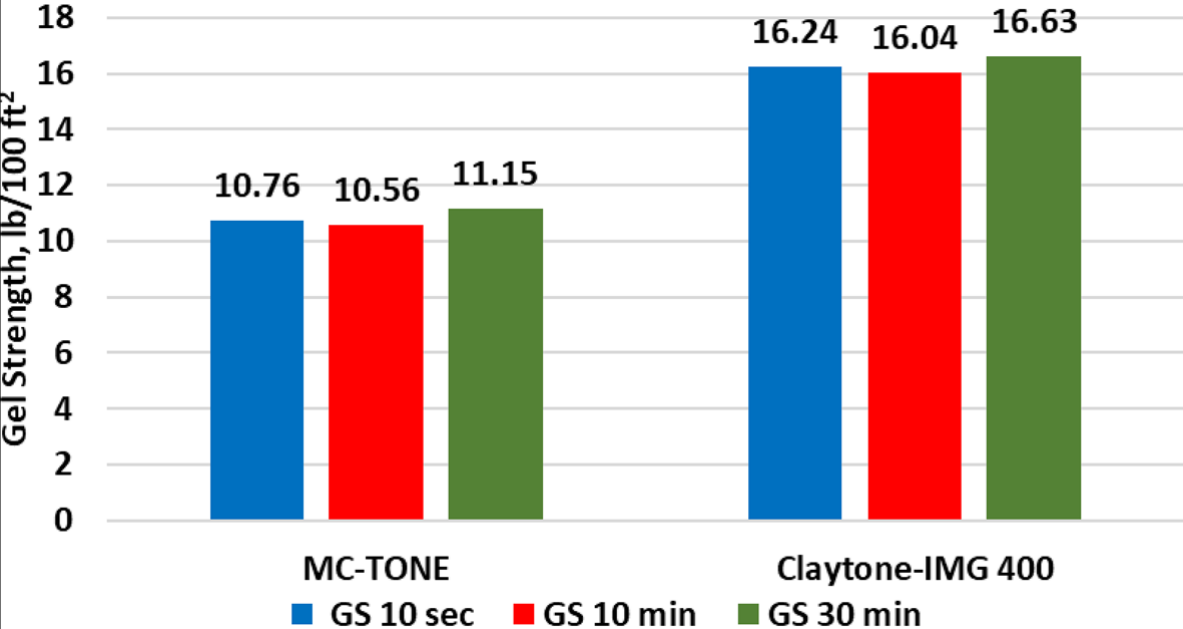
Effect of Claytone-IMG 400 on gel strength at 275 °F.

The effect of Claytone-IMG 400 on the drilling fluid rheology was examined and compared with the drilling fluid formulated using MC-TONE. Figure 12 confirms that Claytone-IMG 400 produces higher shear stress and viscosity in the low shear range. This results in better suspension performance, gelling, and sag. The Bingham plastic model is used to describe the rheological behavior of the two drilling fluid samples with R^2^ values of 0.9773 (Claytone-IMG 400) and 0.9803 (MC-TONE). Several studies have provided support for the applicability of the Bingham plastic model in characterizing the flow properties of OBDFs^10,15,56,60^. Werner et al.^56^ demonstrated the effectiveness of the Bingham plastic model in capturing the viscoelastic properties and flow behavior of OBDFs, particularly under HPHT conditions. In addition, Fakoya and Ahmed^10^ highlighted the suitability of the Bingham plastic model for predicting the apparent viscosity of OBDFs, further emphasizing its relevance in understanding the rheological characteristics of OBDFs. These findings collectively underscore the validity and applicability of the Bingham plastic model in characterizing the flow properties and rheological behavior of OBDFs, thereby establishing its significance in the analysis and optimization of drilling fluid performance. The effect of Claytone-IMG 400 on the drilling fluid rheology is shown in Fig. 13 at 275 °F. The plastic viscosity (PV) was increased by a 30% increment from 22.45 cP for the drilling fluid formulated using MC-TONE to 29.14 cP with Claytone-IMG 400 OC. Claytone-IMG 400 can increase the plastic viscosity of drilling fluid by forming a network structure within the fluid. This network helps to improve the fluid's flow resistance, which can be beneficial for suspending and transporting drill cuttings and maintaining proper hole cleaning. Drilling fluids are typically non-Newtonian and exhibit shear-thinning behavior, meaning their viscosity decreases with increasing shear rate^56^. Claytone-IMG 400 can enhance this shear-thinning behavior, providing a higher plastic viscosity at low shear rates and a lower plastic viscosity at high shear rates. This property can be advantageous for drilling operations, as it allows for better suspension of cuttings and reduced frictional pressure losses in the circulation system. The addition of Claytone-IMG 400 can also improve the thixotropic behavior of drilling fluid, allowing it to thin under shear stress and recover its initial viscosity once the stress is removed. This property can help maintain the fluid's stability and carrying capacity during drilling operations while ensuring that it flows easily through the system when necessary. Furthermore, the yield point increased by a 38% increment from 25.06 to 34.67 lb/100ft^2^ due to the high dispersion of Claytone-IMG 400. This increase in yield point improves the fluid's ability to suspend and transport drill cuttings, which is essential for maintaining proper hole cleaning and preventing sag or settlement of cuttings during drilling operations. Furthermore, a higher yield point can help maintain borehole stability by providing better support to the borehole walls. The addition of Claytone-IMG 400 can improve the drilling fluid's capacity to resist deformation and flow under low-stress conditions, preventing the walls of the borehole from collapsing or sloughing during drilling operations^18^. Also, the yield point plays a role in the formation of an effective filter cake on the borehole walls. A drilling fluid with a higher yield point can create a more stable and uniform filter cake that helps minimize fluid loss and stabilize the borehole. Moreover, Claytone-IMG 400 can enhance the thixotropic behavior of drilling fluid, allowing it to thin under shear stress and quickly recover its initial viscosity once the stress is removed. This property can contribute to the fluid's ability to maintain its yield point and carrying capacity during drilling operations, while also ensuring that it flows easily through the system when necessary^61^. The apparent viscosity (AV) increased by a 33% increment from 34.98 to 46.47 lb/100ft^2^. The YP/PV ratio increased with a 6% increment from 1.12 to 1.19. A higher YP/PV ratio indicates better suspension and carrying capacity for drill cuttings, which is crucial for maintaining hole cleaning and preventing sag or settling of cuttings during drilling operations^2,3^. Figure 14 shows that the high viscosity at a low shear rate of Claytone-IMG 400 enhanced the suspension ability and gelling strengths (GS). The gelling strengths at 10 s, 10 min, and 30 min were increased from 10.76, 10.56, and 11.15 lb/100ft^2^ for the drilling fluid formulated using MC-TONE to 16.24, 16.04, and 16.63 lb/100ft^2^ respectively when using Claytone-IMG 400. A high gelling strength increases the fluid's resistance to flow, particularly when static or under low shear rates, helping to suspend and transport drill cuttings and maintain borehole stability.

### Filtration tests

**Figure 15:**
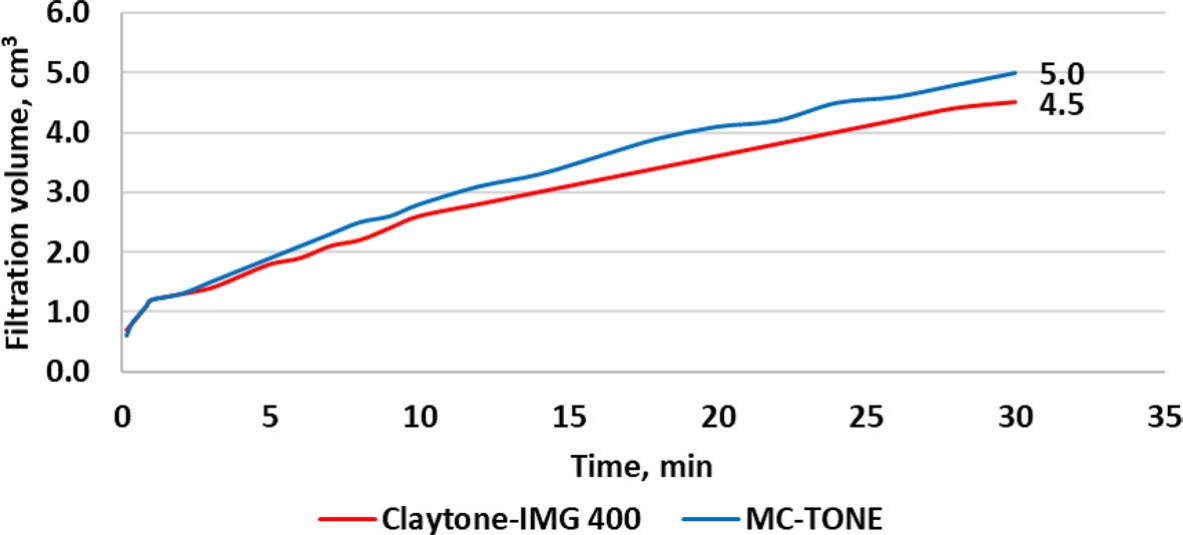
Effect of Claytone-IMG 400 on the filtration volume at 275 °F.

**Figure 16:**
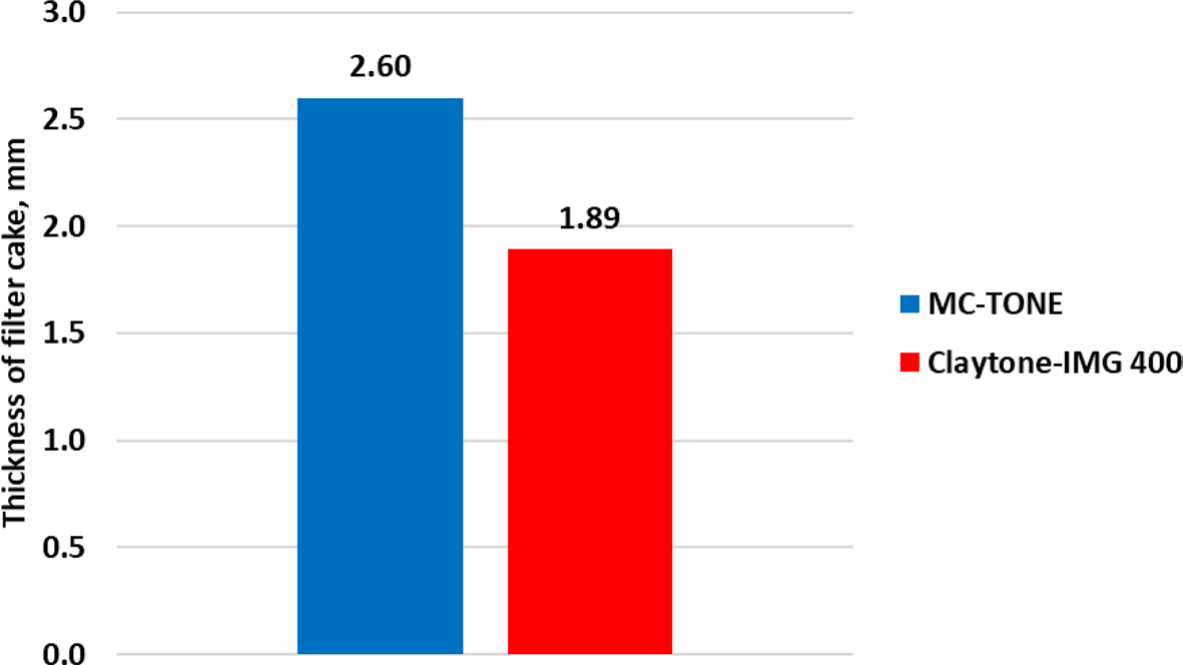
Effect of Claytone-IMG 400 on the filter cake thickness at 275 °F.

The filtration test results showed that Claytone-IMG 400 enhanced the filtration properties. Figure 15 shows a 10% reduction in the filtration volume from 5 to 4.5 cm^3^, while Fig. 16 shows a significant decrease of 37.5% from 2.60 to 1.89 mm in the filter cake thickness. The addition of Claytone-IMG 400 reduced the fluid loss of drilling fluid by increasing its AV and GS. These rheological properties can help create a more effective filter cake, which can better prevent the loss of fluid to the formation during drilling operations. Reduced fluid loss is crucial for maintaining wellbore stability and minimizing formation damage. Also, Claytone-IMG 400 improved the formation of a stable and uniform filter cake on the borehole walls. This enhancement is due to the ability to form a network structure within the fluid, which can help minimize fluid loss and stabilize the borehole. A well-formed filter cake is essential to maintain drilling efficiency and prevent excessive fluid invasion into the formation, which can cause formation damage or reduced productivity.

## Conclusions

A comprehensive experimental study was conducted to evaluate the influence of Claytone-IMG 400 on enhancing the properties of OBDFs at HPHT conditions. A comparative analysis was conducted with MC-TONE, a pre-existing OC, to assess the improvements achieved by Claytone-IMG 400. The outcomes of this study are listed below:

- Claytone-IMG 400 does not affect the drilling fluid density, while it enhances the electrical stability of the invert emulsion with a 9% increment compared to the reference drilling fluid formulated using MC-TONE. This is due to its relatively small particle size and high surface area.
- Claytone-IMG 400 reduces the static and dynamic sag by imparting thixotropic behavior, contributing to the yield stress, promoting stable particle interactions, and adsorbing onto suspended particle surfaces.
- The rheological properties were enhanced with Claytone-IMG 400 as it significantly improved the suspension and gelling capabilities of the drilling fluid. Also, key characteristics, such as PV, YP, and AV were improved by 30%, 38%, and 33% increments respectively.
- The YP/PV ratio increased with a 6% increment from 1.12 to 1.19. This indicates better suspension and carrying capacity for drill cuttings, which is crucial for maintaining hole cleaning and preventing sag or settling of cuttings during drilling operations.
- The gel strength and viscoelastic properties increased with Claytone-IMG 400 which indicated better suspension and hole cleaning performance. Claytone-IMG 400 results in increased complex viscosity, indicating a higher resistance to flow under oscillatory shear.
- The filtration volume was reduced by 10% to 4.5 cm^3^, and the filter cake thickness had a 37.5% reduction from 2.60 to 1.89 mm.

## Data Availability

The datasets used and/or analyzed during the current study are available from the corresponding author upon reasonable request. The results presented in the study are based on the sets of laboratory experiments performed on the oil-based drilling fluid formulation.
